# Nutritional Status and Mediterranean Diet Adherence in Urban Albanian School-Aged Children and Adolescents: A Cross-Sectional Study

**DOI:** 10.3390/children13030398

**Published:** 2026-03-12

**Authors:** Ruden Cakoni, Stefania Moramarco, Angela Andreoli, Jemine Shima, Fjola Kore, Anila Godo, Ersilia Buonomo

**Affiliations:** 1 Faculty of Medicine, Catholic University of Our Lady of Good Counsel, 1026 Tirana, Albania; angela.andreoli@uniroma2.it (A.A.); j.shima7537@stud.unizkm.al (J.S.); a.godo@unizkm.al (A.G.); ersilia.buonomo@uniroma2.it (E.B.); 2 PhD School of Nursing and Public Health, University of Rome Tor Vergata, 00133 Rome, Italy; 3 Department of Biomedicine and Prevention, University of Rome Tor Vergata, 00133 Rome, Italy; stefania.moramarco@uniroma2.it; 4 Department of Systems Medicine, University of Rome Tor Vergata, 00133 Rome, Italy; 5 Primary Health Care Center, 6001 Gjirokaster, Albania; fjolakore@gmail.com

**Keywords:** KIDMED, Albania, gender, adolescents, dietary habits, overweight, urban area

## Abstract

Background: Healthy eating habits during childhood and adolescence are essential to support optimal nutritional status and may influence health in adulthood. This study assessed nutritional status and adherence to the Mediterranean diet (MD) among Albanian students. Methods: A cross-sectional study was conducted on a convenience sample of 388 students aged 9–17 years living in Vlora and Tirana. Data collected included sociodemographic information, anthropometric measurements, and KIDMED index. Factors associated with poor MD adherence were investigated using univariate analysis and multivariable logistic regression (AORs: 95% CIs). Results: Data from 388 students were included in the analysis (mean age: 13.0 ± 1.9 years; 54.1% females). Pre-adolescents (≤13 years) accounted for 53.4% of the sample, while adolescents (>13 years) accounted for 46.6%. Overall, 35% of students were overweight or obese. The mean KIDMED score indicated suboptimal MD adherence (5.5 ± 2.6), with significant differences by sex (females 5.2 ± 2.7 vs. males 5.8 ± 2.5; *p* = 0.03), place of residence (Vlora 5.8 ± 2.5 vs. Tirana 5.2 ± 2.6; *p* = 0.05), and age (≤13 years 6.1 ± 2.3 vs. >13 years 4.8 ± 2.7; *p* < 0.001). Adolescence was the strongest predictor of poor MD adherence (AOR 3.25; 1.96–5.38; *p* < 0.001). Conclusions: The MD is a key dietary pattern for supporting health across the life course. Girls and adolescents showed poorer MD profiles. Further research is needed to clarify the determinants that drive suboptimal dietary behaviors in Albanian youth, in line with growing concerns about the “hidden crisis” of adolescent nutrition.

## 1. Introduction

Eating patterns have a substantial influence on healthy growth and development during childhood and adolescence and are associated with health outcomes later in life [1]. During these formative years, a balanced diet supports adequate nutritional status and may reduce the risk of both malnutrition and non-communicable diseases (NCDs). Nevertheless, the global rise in overweight and obesity among children and adolescents—and its cardiometabolic sequelae—remains a major public health concern [2].

Social epidemiology has consistently linked lower diet quality to demographic characteristics (e.g., age and sex) and socioeconomic conditions (e.g., income and education) [3].

Regarding age, a critical vulnerability emerges during the transition from childhood to adolescence, when diet quality frequently deteriorates. Across countries and regions, adolescent diets are often characterized by low intake of fruits, vegetables, whole grains, lean meats, and low-fat dairy, coupled with high consumption of confectionery, sugar-sweetened beverages, fats, processed meats, refined grains, and ready-to-eat foods [4]. This pattern is particularly concerning because adolescence is a pivotal developmental phase in which individuals gain autonomy and progressively assume responsibility for food choices, culminating in independence from parents or guardians. At the same time, increased physiological requirements for energy and nutrients and still-maturing cognitive and self-regulatory capacities may heighten vulnerability to both undernutrition and overnutrition [5]. In this context, the adoption of unhealthy eating behaviors may contribute to obesity and elevate long-term risk of chronic disease [6]. Importantly, dietary patterns established during adolescence tend to track into adulthood, reinforcing their potential lifelong impact [1].

Gender affects adolescent health and nutrition due to pubertal biological changes, particularly during the transition to secondary school [7].

Beyond individual developmental factors, diet quality is shaped by the broader social environment. Concurrently, ongoing economic transition, industrialization, and globalization have substantially reshaped dietary behaviors, often favoring processed foods high in fats and sugars. These shifts have been especially pronounced in urban environments, where rapid food-system changes and technological transformations have modified both food access and everyday eating practices. Through changing production systems, pricing, trade, and marketing, urbanization and globalization can increase the availability and affordability of non-traditional products, accelerating the transition from traditional food systems to increasingly industrialized food environments [8]. This evolution aligns with the “nutrition transition,” defined by a move from diets centered on staple grains, fruits, and vegetables to patterns increasingly dominated by highly processed packaged foods [9]. Such diets are typically energy-dense and high in nutrients of concern; consequently, the burden of diet-related NCDs—particularly obesity, type 2 diabetes, and cardiovascular disease—has frequently been interpreted as a downstream outcome of these food-system shifts [10].

Within this landscape, the Mediterranean diet (MD) remains one of the dietary models most consistently associated with health protection, including reduced risk of NCDs [11,12]. However, despite its well-documented benefits, MD adherence has declined in multiple settings in recent decades, plausibly driven by the nutrition transition [13]. Therefore, adolescent nutrition remains underprioritized in global health agendas. The Lancet Commission on Adolescent Health and Wellbeing highlighted this gap [14], and adolescent nutrition has been described as a “hidden crisis” [15].

Recent studies have highlighted the importance of investigating determinants of MD adherence also in non-Mediterranean countries, particularly because residing in the Mediterranean region does not necessarily translate into higher compliance among local populations [16]. In Albania, coastal areas largely retain Mediterranean-style eating patterns, with higher consumption of fish and seafood, whereas inland and urbanized areas show greater influence from the nutrition transition, characterized by cereal-based diets, higher intake of meat and processed foods, and lower consumption of fruits and vegetables [17]. However, comprehensive evidence on MD adherence among school-aged populations remains limited, particularly in urban contexts undergoing rapid social and dietary change [18]. Building on a previous pilot study conducted in rural and semi-urban areas [19], we conducted a cross-sectional study in urban settings to capture the potential influence of nutrition transition and urbanization-related lifestyle changes on dietary patterns and nutritional status in younger populations. The primary aim of this study was to explore nutritional status and to assess adherence to the MD among Albanian school-aged children and adolescents using the validated KIDMED questionnaire, and to examine differences in MD adherence and its components by school level. We also aimed to evaluate associations with key sociodemographic factors, identifying predictors of poor dietary habits.

## 2. Materials and Method

### 2.1. Study Design and Population

This cross-sectional study was conducted between March and November 2025 in schools in urban areas in Albania: Tirana (the capital) and Vlora (a coastal city). Specifically, one combined primary and secondary school in Vlora, one lower secondary school in Tirana and one upper secondary school in Tirana were included in the study. A convenience sample of classes from grade 4 to grade 11 was selected. The investigation was conducted during school hours in the classroom in the presence of a teacher. Inclusion criteria were enrollment in grades 4 to 11 in the selected schools, attendance on the day of data collection, and provision of verbal parental consent through the school directory. Exclusion criteria included absence on the assessment day, refusal to participate, incomplete questionnaires or missing anthropometric measurements, and motor disabilities that prevented accurate anthropometric assessment.

### 2.2. Ethical Considerations

Before the data collection, prior contact was established with the school directory of each school. The study’s objectives and methods were shared with the director and the teaching staff, and a copy of the questionnaire was provided in advance. During the in-person meeting, the staff member was provided with sufficient details regarding the study (procedures including anthropometric and dietary habits assessment), and information related to the anonymity of the study was explained. The school administration informed the students and their parents. Consent was obtained from the parents through teachers. Following the acquisition of the consent the data collection commenced. Participation in the study was entirely voluntary. Students who were absent on the assessment day and those who did not wish to participate in the evaluation were not included in the study. The study adhered to the “Helsinki World Medical Association Declaration (1975)—Ethical Principles for Medical Research Involving Human subjects”.

### 2.3. Data Collection, Questionnaire and Measurements

Questionnaires were self-administered to the students by the trained staff members of the Albanian Society of Nutrition Science (ASNS). The team consisted of one PhD candidate and two medical students, who were present throughout the entire process, as well as a medical doctor in Vlora and a teacher in one of the schools of Tirana. At the beginning of the class, researchers explained the main purpose of the study and the study protocol. After the explanation, students completed the questionnaires. The researchers were available to explain or clarify the questions to the participants. The procedure was anonymous.

Data were collected using a printed questionnaire divided into four sections: the first part was for socio-demographic information, the second part for anthropometric measurements, and the third part for dietary habits. Each participant provided initials instead of their full name to ensure confidentiality. The questionnaire was administered in the Albanian language. The instruments were translated by two independent bilingual Albanian–Italian academicians, and back-translation was performed to ensure accuracy and semantic equivalence. Sociodemographic characteristics included variables such as gender, age, and place of residence.

Anthropometric measurements included weight and height and were taken on site. Body weight and height were measured using the same calibrated scale and stadiometer for all participants to ensure measurement consistency. Students were assessed wearing light clothing and without shoes. Weight measurements were accurate to ±100 g, and height measurements to ±1 cm. Body mass index (BMI) was calculated according to age- and sex-specific international WHO standards BMI-for-age Z-scores (BAZ) and height-for-age Z scores (HAZ) were calculated using WHO Anthro Software version 3.3.2 [20]. Nutritional status was classified according to the WHO Child Growth Standards [21] as: overweight: BAZ > +1 SD; obesity: BAZ > +2 SD; underweight: BAZ < −2 SD; stunting (chronic malnutrition); HAZ < −2 SD.

Adherence to the Mediterranean Diet (MD) was assessed using the Mediterranean Diet Quality Index for Children and Adolescents (KIDMED) [22]. The KIDMED questionnaire consists of 16 questions with two possible answers, assigned −1 or 1, with a final score from 0 to 12. Based on the final score, adherence to the Mediterranean diet was classified as: ≤3 poor MD adherence; 4–7 intermediate MD adherence; ≥8 high or optimal MD adherence. The Italian version of the KIDMED questionnaire was independently translated into Albanian by two bilingual academicians. Back-translation was used to confirm consistency with the original instrument.

### 2.4. Statistical Analysis

The database with anonymized data was analyzed by Statistical Package for Social Sciences (SPSS version 26.0, IBM, Somers, NY, USA). Analysis was conducted for totals and by grouping as for sex (M vs. F), age (younger than 13 years vs. 13 years or older), and geographic area (Tirana vs. Vlora). Continuous variables were reported as means with standard deviations (SD), while categorical variables were presented as numbers and percentages. The independent samples t-test was used for comparisons between groups, while the chi-square test was applied to compare proportions. Odds ratios (ORs) with a 95% confidence interval (95% CI) were calculated to identify factors influencing dietary patterns. Statistical significance was set at *p*-value ≤ 0.05 and was based on two-sided test. Univariate and multivariate logistic regression were performed to identify which of the three variables (sex, age, and geographic area) had the greatest influence on low adherence to the Mediterranean diet. Given the presence of multiple independent variables, a forward stepwise regression approach was applied.

## 3. Results

A convenience sample of 388 students, of which 210 females (54.1%) and 178 males (45.9%), attending secondary schools was selected. Specifically, 231 in Tirana (59.5%) and 157 (40.5%) in Vlora. The mean age was 13.3 years ± 1.9 SD, and a range from 9 to 17 years. Slightly more than half of participants were pre-adolescents (≤13 years: *n* = 207; 53.4%), while 46.6% were older than 13 years (*n* = 181). According to academic grade, 17 students (4.4%) were in the 4th grade, 16 (4.1%) in the 5th grade, 100 (25.8%) in the 6th grade, 66 (17.0%) in the 7th grade, 30 (7.7%) in the 8th grade, 116 (29.9%) in the 9th grade, 21 (5.4%) in the 10th grade, and 22 (5.7%) in the 11th grade. Regarding place of residence, 59.5% of participants lived in Tirana (*n* = 231) and 40.5% in Vlora (*n* = 157) (Table 1).

Table 2 summarizes anthropometric indicators and nutritional status across sex, age group, and place of residence. Males presented higher HAZ and BAZ values than their counterparts (HAZ: 0.87 ± 1.24 vs. 0.59 ± 1.08; *p* = 0.01; BAZ: 0.78 ± 1.19 vs. 0.28 ± 1.06; *p* < 0.001), while BMI was only marginally higher in males (21.12 ± 3.87 vs. 20.39 ± 3.53; *p* = 0.05). Adolescents (>13 years) had substantially higher BMI than pre-adolescents (≤13 years) (21.76 ± 3.61 vs. 19.82 ± 3.56; *p* < 0.001). Conversely, HAZ and BAZ were higher in the younger group (1.00 ± 1.24 vs. 0.40 ± 0.98; *p* < 0.001; 0.61 ± 1.20 vs. 0.39 ± 1.07; *p* = 0.05). Considering place of residence, students living in Tirana had a higher mean weight and height than those in Vlora (55.62 ± 13.01 vs. 53.39 ± 16.02 kg; *p* = 0.008; 161.81 ± 9.40 vs. 160.86 ± 13.69 cm; *p* < 0.001), whereas BMI, HAZ, and BAZ did not differ significantly.

Regarding nutritional status, a high prevalence of overweight and obesity was observed in the overall sample, affecting over thirty percent of participants. Conversely, underweight and stunting were rare (≤1.8% and 1.0%, respectively) and did not differ significantly across subgroups. The prevalence of obesity and overweight was markedly higher among males (18.0% vs. 3.8%, *p* < 0.001; 29.8% vs. 20.0%, *p* = 0.01), while no significant differences were detected between age groups; however, normal weight was more frequent among adolescents (69.1% vs. 58.5%, *p* = 0.02). By place of residence, Tirana showed the highest prevalence of overweight students (27.7% vs. 19.7%, *p* = 0.04), whereas normal weight was higher in Vlora (69.4% vs. 59.3%, *p* = 0.02).

Table 3 shows the answers to the KIDMED questionnaire. The overall mean KIDMED score was 5.5 ± 2.6 SD, indicating an intermediate level of adherence to the MD. There were statistically significant differences between pre-adolescents and adolescents (6.1 ± 2.3 SD vs. 4.8 ± 2.7 SD, *p* < 0.001), females and males (5.2 ± 2.7 SD vs. 5.8 ± 2.5 SD, *p* = 0.03) and place of residence (5.8 ± 2.5 SD vs. 5.2 ±2.6 SD, *p* = 0.05) (Table 3).

When examining individual KIDMED items, daily consumption of a first serving of fruit and vegetables was reported by approximately 70% of participants. However, intake of a second daily serving was substantially lower, declining to about 50% for fruit and 30% for vegetables. Regular fish consumption was reported by fewer than half of the sample, and similarly low proportions were observed for consuming pulses more than once per week and for regular nut intake. In contrast, olive oil use was almost universal (>95%). Breakfast habits were suboptimal: nearly one third of students reported not eating breakfast regularly, and only slightly more than half consumed a dairy product at breakfast. This pattern was consistent with the proportion reporting daily consumption of two yogurts and/or some cheese. Finally, almost half of the sample reported eating sweets or candy several times per day, and nearly one third reported visiting a fast-food restaurant more than once per week.

After stratifying by groups, compared to males, females were less likely to frequently consume fast food (OR 0.4; 95% CI 0.3–0.6; *p* < 0.001), but were more likely to skip breakfast (OR 3.0; 95% CI 1.9–4.9; *p* < 0.001), to not eat daily fruit (OR 2.6; 95% CI 1.2–3.4; *p* = 0.005), to not consume dairy products at breakfast (OR 2.3; 95% CI 1.5–3.5; *p* < 0.001), and to consume sweets and candy several times every day (OR 1.6; 95% CI 1.1–2.3; *p* = 0.019). Compared with adolescents, pre-adolescents were significantly less likely to frequently consume fast food (OR 0.4; 95% CI 0.3–0.7; *p* < 0.001), to consume sweets (OR 0.5; 95% CI 0.3–0.8; *p* = 0.002), to skip breakfast (OR 0.2; 95% CI 0.1–0.4; *p* < 0.001), to not have daily fruit consumption (OR 0.4; 95% CI 0.3–0.7; *p* = 0.001), and not consume a dairy product at breakfast (OR 0.4; 95% CI 0.2–0.5; *p* < 0.001). Regarding geographic area, students living in Tirana had higher odds of skipping breakfast (OR 1.9; 95% CI 1.2–3.2; *p* = 0.002) and of reporting lower regular consumption of fish (OR 2.2; 95% CI 1.4–3.3; *p* < 0.001) compared with those living in Vlora.

**Figure 1:**
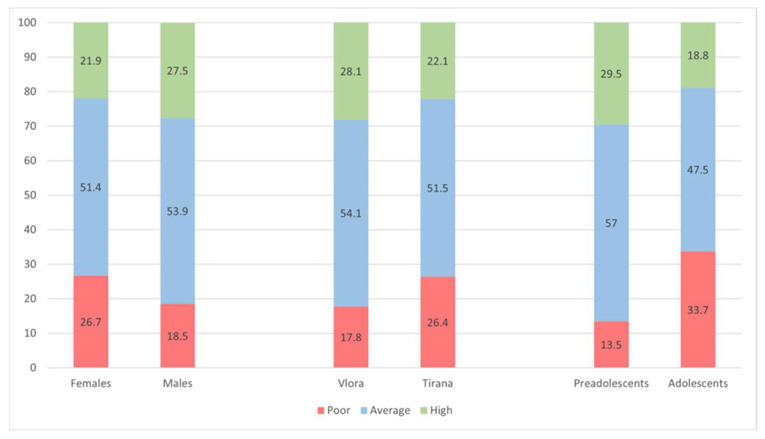
Adherence to the Mediterranean Diet (MD) by sex, age group, and geographic area.

Regarding the Mediterranean diet, 22.9% of the overall sample fell into the poor KIDMED category, 52.6% showed an intermediate MD profile, and 24.5% achieved a high MD score. When results were stratified by sex, geographic area, and age, all the three categories were significantly associated with adherence to the Mediterranean diet (Figure 1). Girls more frequently displayed poor MD scores than boys (26.7% vs. 18.5%), whereas high MD scores were more common among boys (27.5% vs. 21.9%). By residence, students living in Tirana were more likely to present poor MD adherence than those from Vlora (26.4% vs. 17.8%). Age-related differences were pronounced: adolescents had a substantially higher prevalence of low MD adherence compared with pre-adolescents (33.7% vs. 13.5%), while a high MD score was more frequent in the younger group (29.5% vs. 18.8%).

Binary logistic regression (Table 4) indicated that, among the three variables examined, age was most strongly associated with low adherence to the MD. In particular, being older than 13 years emerged as the strongest predictor of poor MD compliance (OR 3.25; 95% CI 1.96–5.38; *p* < 0.001).

## 4. Discussion

The Mediterranean diet is recognized for its protective effects on long-term cardiometabolic health [11,12,23]. Because eating habits are established progressively through physiological, cognitive, and social development, early, structured initiatives can help consolidate healthier and more sustainable dietary practices [24,25]. Although adolescence is a period of rapid physical and cognitive development associated with increased nutritional needs—second only to infancy and therefore described as the second major “developmental period” [26]—the Lancet Commission highlighted that adolescents are often overlooked in global health policies and called for greater investment to protect their health and future well-being [14]. The main aim of this study was to assess the nutritional status of school-aged children living in urban areas of Albania and to examine differences in Mediterranean diet adherence and its components across sociodemographic groups. To the best of our knowledge, this is among the few studies to investigate MD-related dietary behaviors in both pre-adolescents and adolescents in Albanian settings. Our findings indicate that poor diet quality was present in both pre-adolescents and adolescents, in line with European evidence [27,28,29,30]. Approximately one quarter of the overall sample fell into the low KIDMED category, and this proportion increased to over 30% among adolescents, approaching the level reported by Novak et al. According to them, low KIDMED scores were more frequent among girls [16]. This trend was also confirmed in our previous investigation conducted in rural areas in Albania [19], as well as in a study conducted in Greece [31]. Our findings indicate an inverse association between age and Mediterranean diet adherence, with lower MD scores observed among older students [19,30], despite other data in the literature reporting a different trend [27,28]. Increased independence in daily routines and food choices, reduced parental supervision, and a tendency to distance themselves from family models—including eating practices—may contribute to a shift away from healthier habits [32,33] when parental control could matter less for healthy dietary habits [34]. When considering specifically the KIDMED items, daily breakfast consumption was more frequent among males and younger participants, consistent with findings from a survey of Albanian schoolchildren [35]. Skipping breakfast among females has been an eating attitude confirmed in previous studies conducted on school-aged children in different contexts [28,36,37]. Similarly, in our sample, fruit and/or vegetable consumption was higher among younger children, consistent with previous studies showing that older age is associated with lower daily fruit intake [3]. However, unlike Stefa et al., we observed lower fruit and/or vegetable consumption among females. In line with the same authors, males in our study reported a lower daily intake of sweets; in contrast, the age-related pattern differed, as younger participants in our sample reported this behavior less frequently [35]. Regarding fast food, our findings were consistent with those of Archero et al., with males reporting more frequent visits per week [29]. We also observed an opposite age pattern, as in in our sample adolescents reported going to fast food more often than younger participants.

A notable difference emerged by area of residence: students living in Tirana had lower overall KIDMED scores than those living in Vlora. This may be partly explained by higher fish consumption in the coastal setting [19], suggesting that dietary profiles may vary across Albanian contexts and highlighting the need for broader surveillance. We also observed a high burden of increased body weight in the total sample, with the combined prevalence of overweight and obesity exceeding that reported in some international adolescent samples in non-Mediterranean European countries [16]. Our sex-specific findings were consistent with theirs, as males showed higher BMI values, however no assessment of body composition or pubertal status was performed [16,38]. Adherence to Mediterranean Diet tended to decrease with increasing age, consistent with our previous findings [19]. The findings of the current study should be interpreted in light of some limitations. First, due to its cross-sectional design, causal inferences cannot be made. Second, Mediterranean diet adherence was assessed using a self-reported questionnaire; therefore, participants may have underreported or overreported their eating behaviors. Third, the use of a relatively small convenience sample limits representativeness across Albania; therefore, the findings should be interpreted with caution. Fourth, we did not collect information on physical activity, sedentary behaviors, psychological factors, or socioeconomic status, all of which may influence MD-related dietary behaviors [16]. However, the amount of information bias is comparable to that of similar epidemiological studies, as the self-reported questionnaire has been extensively validated in previous research among schoolchildren [22].

### Limitations

This study has several limitations that should be acknowledged. The cross-sectional design of the study does not allow us to determine cause-and-effect relationships; therefore the findings should be interpreted with caution. Moreover, the absence of a control group and longitudinal follow-up further limits the strength and robustness of the conclusions. Second, the use of a convenience sample may limit the representativeness of the study population. Although participants were recruited from major urban centers, the sample does not reflect all geographic regions of Albania; consequently, the generalizability of the findings may be limited. Third, the statistical analysis did not adjust for potential confounding variables such as physical activity levels, access to social media, and socioeconomic status, which may have influenced the observed associations. As data were collected through self-reported questionnaires, some self-report bias cannot be entirely ruled out, even though the same questionnaire has been validated in our previous paper among schoolchildren in rural areas [19] which supports the reliability and comparability of the collected data. Considering these limitations, the findings of this study should not be generalized to all Albanian schoolchildren or the broader population. Further longitudinal and nationally representative studies are warranted to confirm and expand upon these results.

## 5. Conclusions

The Mediterranean diet is a key dietary pattern for supporting health across the life course. School-aged children and adolescents represent a priority group for health promotion, as dietary behaviors developed during these formative years shape growth, development, and long-term health trajectories. Improving nutrition in this life stage is a sound investment in future population health, whereas persistent unhealthy eating patterns may carry lasting consequences for cardiometabolic risk and well-being. In our study, girls, adolescents, and students living in the capital (Tirana) showed poorer MD profiles and specific gaps in dietary habits, with adolescents emerging as the most vulnerable group. These findings can inform public health strategies aimed at reducing dietary inequalities by prioritizing at-risk subgroups. Future research should incorporate additional lifestyle determinants and support context-specific interventions and policies to strengthen healthy eating behaviors in these populations, an important step toward advancing the Global Nutrition Targets and sustaining momentum in the final push toward the 2030 Agenda for Sustainable Development [39].

## Figures and Tables

**Table 1:** Socio-demographic characteristics of the total sample.

Variables	Value
Female, *n*. (%)	210 (54.1)
Age (min-max)	9–17 years
Age, mean years ± SD	13.3 ± 1.9
Age groups, *n*. (%)	
≤13 years	207 (53.4)
>13 years	181 (46.6)
School grade, *n*. (%)	
IV	17 (4.4)
V	16 (4.1)
VI	100 (25.8)
VII	66 (17.0)
VIII	30 (7.7)
IX	116 (29.9)
X	21 (5.4)
XI	22 (5.7)
Place of Residence, *n*. (%)	
Tirana	231 (59.5)
Vlora	157 (40.5)

**Table 2:** Anthropometric characteristics by totals, gender and age group.

Variables	Totals (*n*.388)	Female (*n*.210)	Males (*n*.178)	*p*-Value	Pre-adolescents ≤ 13 (*n*.207)	Adolescents > 13 (*n*.181)	*p*-Value
Weight (kg), mean ± SD	54.72 ± 14.33	52.61 ±11.75	57.20 ±16.56	0.002	48.75 ± 12.31	61.54 ± 13.42	<0.001
Height (cm), mean ± SD	161.43 ± 11.33	160.04 ±8.62	163.07± 13.70	0.008	155.99 ± 10.60	167.64 ± 8.62	<0.001
Body Mass Index (BMI), mean ± SD	20.34 ± 3.71	20.39 ± 3.53	21.12± 3.87	0.05	19.82 ± 3.56	21.76 ± 3.61	<0.001
Height for Age Z-score (HAZ), mean ± SD	0.7 ± 1.17	0.59 ± 1.08	0.87 ± 1.24	0.019	1.00 ± 1.24	0.40 ± 0.98	<0.001
BMI for Age Z-score (BAZ), mean ± SD	0.53 ± 1.15	0.28 ± 1.06	0.78 ± 1.19	<0.001	0.61 ± 1.20	0.39 ± 1.07	0.05
Nutritional status, *n* (%)							
Obesity	40 (10.3)	8 (3.8)	32 (18.0)	<0.001	26 (12.6)	14 (7.7)	NS
Overweight	95 (24.5)	42 (20.0)	53 (29.8)	0.01	56 (27.1)	39 (21.5)	NS
Normal weight	246 (63.4)	155 (73.8)	91 (51.1)	<0.001	121 (58.5)	125 (69.1)	0.02
Underweight	7 (1.8)	5 (2.4)	2 (1.1)	NS	4 (1.9)	3 (1.7)	NS
Stunting	4 (1.0)	2 (1.0)	2 (1.1)	NS	2 (1.0)	2 (1.1)	NS

**Table 3:** Distribution of questionnaire responses by age and gender.

			Gender					Age				
	Total (*n*.388)		Female (*n*.210)		Males (*n*.178)		*p*-Value	Pre-adolescents ≤ 13 (*n*.207)		Adolescents > 13 (*n*.181)		*p*-Value
Total KIDMED score, mean ± SD	5.5 ± 2.6		5.2 ± 2.7		5.8 ± 2.5		0.03	6.1 ± 2.3		4.8 ± 2.7		<0.001
KIDMED items	Yes, n.(%)	No, n.(%)	Yes, n.(%)	No, n.(%)	Yes, n.(%)	No, n.(%)		Yes, n.(%)	No, n.(%)	Yes, n.(%)	No, n.(%)	
Takes a fruit every day	308 (79.4)	80 (20.6	156 (74.3)	54 (25.7)	152 (85.4)	26 (14.6)	0.005	177 (85.5)	30 (14.5)	131 (72.4)	50 (27.6)	0.001
Has a second fruit every day	195 (50.3)	193 (49.7)	101 (48.1)	109 (51.9)	94 (52.8)	84 (47.2)	NS	106 (51.2)	101 (48.8)	89 (49.2)	92 (50.8)	NS
Has fresh or cooked vegetables regularly, once a day	272 (70.1)	116 (29.9)	150 (71.4)	60 (28.6)	122 (68.5)	56 (31.5)	NS	150 (72.5)	57 (27.5)	122 (67.4)	59 (32.6)	NS
Has fresh or cooked vegetables more than once a day	117 (30.2)	271 (69.8)	62 (29.5)	148 (70.5)	55 (30.9)	123 (69.1)	NS	66 (31.9)	141 (68.1)	51 (28.2)	130 (71.8)	NS
Consumes fish regularly (at least 2–3 times per week)	161 (41.5)	227 (58.5)	86 (41.0)	124 (59.0)	75 (42.1)	103 (57.9)	NS	92 (44.4)	115 (55.6)	69 (38.1)	112 (61.9)	NS
Goes to a fast-food restaurant more than once per week	110 (28.4)	278 (71.6)	42 (20.0)	168 (80.0)	68 (38.2)	110 (61.8)	<0.001	42 (20.3)	165 (79.7)	68 (37.6)	113 (62.4)	<0.001
Likes pulses and eats them more than once per week	181 (46.6)	207 (53.4)	88 (41.9)	122 (58.1)	93 (52.2)	85 (47.8)	0.027	112 (54.1)	95 (45.9)	69 (38.1)	112 (61.9)	0.001
Consumes whole-grain pasta or whole-grain rice almost every day (5 or more times per week)	168 (43.3)	220 (56.7)	85 (40.5)	125 (59.5)	83 (46.6)	95 (53.4)	NS	79 (38.2)	128 (61.8)	89 (49.2)	92 (50.8)	0.019
Has whole cereals or whole-grains (whole-meal bread, etc.) for breakfast	254 (65.5)	134 (34.5)	136 (64.8)	74 (35.2)	118 (66.3)	60 (33.7)	NS	147 (71.0)	60 (29.0)	107 (59.1)	74 (40.9)	0.009
Consumes nuts regularly (at least 2–3 times per week)	183 (47.2)	205 (52.8)	107 (51.0)	103 (49.0)	76 (42.7)	102 (57.3)	NS	103 (49.8)	104 (50.2)	80 (44.2)	101 (55.8)	NS
Uses olive oil at home	371 (95.6)	17 (4.4)	203 (96.7)	7 (3.3)	168 (94.4)	10 (5.6)	NS	199 (96.1)	8 (3.9)	172 (95.0)	9 (5.0)	NS
Skips breakfast	116 (29.9)	272 (70.1)	84 (40.0)	126 (60.0)	32 (18.0)	146 (82.0)	<0.001	35 (16.9)	172 (83.1)	81 (44.8)	100 (55.2)	<0.001
Has a dairy product for breakfast (yoghurt, milk, etc.)	214 (55.2)	174 (44.8)	96 (45.7)	114 (54.3)	118 (66.3)	60 (33.7)	<0.001	138 (66.7)	69 (33.3)	76 (42.0)	105 (58.0)	<0.001
Has commercially baked goods or pastries for breakfast	106 (27.3)	282 (72.7)	53 (25.2)	157 (74.8)	53 (29.8)	125 (70.2)	NS	56 (27.1)	151 (72.9)	50 (27.6)	131 (72.4)	NS
Eats two yoghurts and/or some cheese (40 g) daily	220 (56.7)	168 (43.3)	116 (55.2)	94 (44.8)	104 (58.4)	74 (41.6)	NS	114 (55.1)	93 (44.9)	106 (58.6)	75 (41.4)	NS
Eats sweets and candy several times every day	178 (45.9)	210 (54.1)	107 (51.0)	103 (49.0)	71 (39.9)	107 (60.1)	0.019	80 (38.6)	127 (61.4)	98 (54.1)	83 (45.9)	0.002

**Table 4:** Logistic regression analysis of factors associated with low adherence to the Mediterranean diet.

	Univariate Analysis		Multivariate Analysis	
Variable	OR (95%CI)	*p*-Value	AOR (95%CI)	*p*-Value
Gender (female)	1.22 (1.00–1.48)	<0.001		
Age (adolescent)	1.71 (1.40–2.08)	<0.001	3.25 (1.96–5.38)	<0.001
Geographic area (Tirana)	1.20 (1.01–1.43)	0.031		

## Data Availability

The data presented in this study are available on request from the corresponding author due to privacy and ethical reasons.

## Abbreviations

The following abbreviations are used in this manuscript:

MD	Mediterranean Diet
KIDMED	Mediterranean Diet Quality Index in Children and Adolescents
AOR	Adjusted odds ratio
ORs	Odds Ratios
NCDs	Non-communicable diseases
CI NS	Confidence interval Non-Significant
CVDs	Cardiovascular diseases
GBD	Global Burden of Disease
WHO	World Health Organization
ASNS	Albanian Society of Nutrition Science
BMI	Body mass index
BAZs	BMI for age Z scores
HAZs	Height-for-age Z scores
SPSS	Statistical Package for Social Sciences
SDs	Standard Deviations
